# Characterizing the chicken gut colonization ability of a diverse group of bacteria

**DOI:** 10.1016/j.psj.2022.102136

**Published:** 2022-08-15

**Authors:** Binita Poudel, Naama Shterzer, Yara Sbehat, Nir Ben-Porat, Michal Rakover, Ron Tovy-Sharon, Dvora Wolicki, Stav Rahamim, Enav Bar-Shira, Erez Mills

**Affiliations:** Department of Animal Science, Robert H. Smith Faculty of Agriculture, Food, and Environment, The Hebrew University of Jerusalem, Rehovot 7610001, Israel

**Keywords:** chicken, probiotics, colonization, microbiome, lactobacillus

## Abstract

The development of probiotics for chickens is a rapidly expanding field. The main approach to probiotics is to administer the probiotic strain throughout the bird's life, usually through incorporation in the feed. However, probiotics which would utilize bacterial strains capable of permanently colonizing the gut after a single exposure are likely to have a greater impact on the developing gut community as well as on the host, would simplify probiotic use and also reduce costs in an industrial setting. Finally, very limited and conflicting information about the colonization ability of different bacterial strains has been reported. Here we report 2 colonization experiments using 14 different bacterial strains from diverse phylogenetic groups. In both experiments, groups of chicks were orally inoculated on the day of hatch with different bacterial strains that had been previously isolated from adult heavy breeders. In the first experiment, colonization of the bacterial strains in broiler chicks was determined 7 d after treatment. In the second experiment, colonization was followed in layer chicks until d 17. Ten of the bacterial strains, including Lactobacillales and Bacteroidales strains, were able to colonize chicks after a single exposure for the duration of the experiment. For a few of these strains, exposure had little effect compared to non-treated chicks due to natural background colonization. Only 4 strains failed to colonize the chicks. Moreover, it is shown that fecal samples are useful to identify and provide a dynamic view of colonization. We further analyzed the effects of artificial colonization on microbiota composition. Some of the strains used in this research were found to reduce Enterobacteriaceae family abundance, implying that they might be useful in reducing relevant pathogen levels. To conclude, our results show that the development of single exposure based probiotics is possible.

## INTRODUCTION

The gut microbiota performs important functions for the host, including protecting the host against gut pathogens and assisting in the digestion of plant derived nutritional fibers that the host cannot digest on its own (Sommer and Bäckhed, 2013; Oakley et al., 2014; Borda-Molina et al., 2018). For these reasons, a lot of effort is applied into research aimed at improving the function of the chicken gut microbiota. A major approach for attempting to improve the function of the gut microbiota is the use of probiotics (Cisek and Binek, 2014; Oakley et al., 2014; Syngai et al., 2016; Suez et al., 2019). Probiotics are live microorganisms which are ingested with the aim of modulating the function of the microbiota. Most research on probiotics is focused on Lactobacillales strains (Suez et al., 2019). This is due to historical-economic reasons. Namely, because Lactobacillales strains are already approved for use, probiotic formulations using them do not need to overcome regulatory barriers, while probiotic formulations containing unapproved bacterial strains do. However, in recent years, exploration of other components of the gut community as probiotics, including *Bifidobacteria, Bacteroides, Bacillus*, and *Clostridium* strains has been expanding (Yang et al., 2012; Kubasova et al., 2019; Tan et al., 2019; Leão et al., 2021).

There are two general approaches to the introduction of the probiotic strains. One is continued exposure throughout the life of the chicken, usually by incorporating the probiotic strain into the feed. This approach is widely used, and even in attempts to expose chicks to probiotics earlier by in-ovo application, the probiotic was added in the feed (de Oliveira et al., 2014). Continuous administration has the advantage that even if the probiotic strain fails to colonize the chicken, it may still have the desired effect by maintaining the level of the probiotic. However, a major disadvantage is that the probiotic strain must survive both the feed and the acidic environment of the stomach, so high levels of probiotics must be administered continuously. A second approach to probiotics is to use a bacterial strain that can colonize the host. Indeed, the application of complex gut content samples from adult birds protects chicks from *Salmonella* infection through a single application (Varmuzova et al., 2016; Litvak et al., 2019). Furthermore, a single treatment of newly hatched chicks with adult cecal contents resulted in a change in host cecal protein expression over the 45 d of the experiment (Volf et al., 2016). One advantage of utilizing a strain capable of colonization is that a single treatment may result in a continuous effect of the probiotic in the chicken gut. The requirement for a single exposure reduces costs, simplifies delivery, and allows for the future development of probiotics based on bacteria with more complicated growth requirements, such as anaerobes. A second advantage is that strains that can colonize the gut can likely be administered at lower doses than strains that cannot do so, as they will expand their numbers once they arrive and colonize the gut. Finally, bacteria which actively colonize chicks are likely to spread through the flock and affect chicks in which initial colonization failed.

Most publications investigating the effects of probiotics fail to report the presence and relative abundance of the administered strains in fecal or internal samples of treated animals, and information regarding the ability of probiotic strains to colonize the host in the literature is generally lacking (Suez et al., 2019). Thus, it is possible that studies may have missed the fact that the first treatment was sufficient, and that further treatment was of little value. Furthermore, the identification of a bacterial strain with a positive effect on the host which cannot colonize the host and requires continued administration to achieve the desired effect, may lead to a search of similar strains, which can colonize the host, resulting in an improved probiotic. For example, if a probiotic based on *Ligilactobacillus salivarius* was shown to have a positive effect on broilers, it is possible that a related chicken adapted strain would have a stronger effect due to its ability to colonize the chicken. Additionally, understanding the colonization ability of different bacterial phylogenetic groups has multiple basic and applied implications. For example, characterization of colonization ability could reveal unoccupied gut niches. Open niches are available for colonization by pathogens or may be utilized to introduce beneficial community members. Similarly, understanding which niches in newly hatched chicks take time to be occupied may help identify deficiencies in the development of gut microbiota functions.

A notable exception to the general lack of knowledge on bacterial colonization ability are 2 recently published studies. The first, characterized the ability of a diverse set of 76 bacterial strains originating from the chicken cecum to colonize newly hatched layers (Kubasova et al., 2019). That study found that many of the examined bacteria were able to colonize the ceca of newly hatched layer chicks up to 7 d of age. However, Kubasova et al. were unable to detect colonization by Firmicutes other than members of class Negativicutes, which is surprising since this includes Lactobacillales strains commonly used as probiotics. The second, studied the ability of a synthetic community compromising 9 strains of diverse phylogeny to colonize the gut of a layer line (Zenner et al., 2021). That study found only 2 of the 9 strains were able to colonize chicks. Here, we extended the colonization characterization in broilers until d 7 of life and in layers until d 17, examined colonization by multiple bacterial strains in both the cecum and jejunum, the usefulness of fecal samples to characterize colonization, and determined the effect of colonization on gut microbiota composition.

## MATERIALS AND METHODS

### Animals

All animal trials were conducted in accordance with the guidelines of the National Council for Animal Experimentation and were subjected to approval by the Hebrew University of Jerusalem's Ethics committee, approval No. AG-20-16070-3. For the first experiment fertilized eggs of the Ross broiler breed were used. For the second experiment fertilized eggs of the Lohmann layer breed were used.

### Egg Incubation

Before incubating the eggs, the egg incubators (MARU 190, Rcom, Korea) were thoroughly cleaned with 0.25% Virocid (CID LINES, Belgium) for disinfection. Eggs were brought from local hatcheries and incubated for 21 d at 37.8°C and 58% relative humidity. The eggs were automatically rotated every hour. Candling of the eggs was done on d 10 to check the viability of the eggs. On d 18 the rotator was turned off. Chicks were hatched in special hatching trays, cleaned with 0.25% Virocid, and placed inside the incubators. Chicks were hatched over 24 h and treated as a group at the same time.

### Bacteria and Chick Inoculation

All of the bacteria used in these 2 experiments were isolated by us as part of an ongoing isolation effort aimed at creating a collection of chicken gut isolates. Isolation was performed on a cecum sample of a 55-wk-old healthy broiler breeder of the Ross breed grown at the Faculty of Agriculture hen house. This bird originated from a commercial operation and was brought to our facility at the age of 37 wk. Initial isolation was on YCFA (Browne et al., 2016) or MRS media (de Man, Rogosa and Sharpe) (Corry et al., 2003), at 37°C, in aerobic or anaerobic conditions (5% H_2_, 20% CO_2_, 75% N_2_). Phylogenetic information based on the 16S rRNA gene as well as growth conditions for these strains are found in Supplementary Table S1. For both experiments, all the bacteria were grown as specified in Supplementary Table S1. Monocultures were grown from a frozen stock in 5 mL of media for 18 h at 37°C. Aerobic bacteria were grown in regular atmospheric conditions in a shaker incubator (250 rpm). Anaerobes were grown in anaerobic conditions (75% N_2_, 20% CO_2_, 5% H_2_) without shaking. After growth, cultures were centrifuged (5430R, Eppendorf AG, Germany) at 3,000 RCF for 10 min, the supernatant was discarded and pellets were resuspended in PBS. The resuspended pellets were centrifuged at 12,000 RCF for 2 min and supernatant was discarded and pellets were resuspended in PBS a second time. Each culture was diluted with PBS to a final concentration of OD_600_ = 1. Newly hatched chicks were treated before any water or food was given. Each treated chick was treated with just one strain. All control and treatment chicks received 200 µL of PBS or bacterial inoculum respectively. A 200 µL pipettor with a 200 µL tip was used to gently open the chick's beak and slowly dispense the culture into the chick's mouth.

### First Experiment - Broilers

For the first experiment 72 newly hatched broiler chicks were randomly allocated into 8 groups: 1) Control treated with PBS only (n = 9), 2) *Phocaeicola plebeius* Yara004 (n = 9), 3) *Phocaeicola salanitronis* Yara001 (n = 9), 4) *Massiliomicrobiota timonensis* Yara022 (n = 9), 5) *Ruminococcus torques* Yara008 (n = 9), 6) *Limosilactobacillus reuteri* Yara020 (n = 9), 7) *Ligilactobacillus salivarius* Yara018 (n = 9), 8) *Pediococcus pentosaceus* Yara000 (n = 9). This experiment lasted 7 d.

### Second Experiment - Layers

For the second experiment 61 newly hatched layer chicks were randomly allocated into 10 groups: 1) Control treated with PBS only (n = 7), 2) *Phocaeicola coprophilus* Yara003 (n = 6), 3) *Megamonas rupellensis* Yara012 (n = 6), 4) *Limosilactobacillus vaginalis* Yara017 (n = 6), 5) *Limosilactobacillus reuteri* Yara020 (same strain as in the first experiment) (n = 6), 6) *Escherichia fergusonii* Yara015 (n = 6), 7) *Ligilactobacillus salivarius* Yara018 (same strain as in the first experiment) (n = 6), 8) unclassified Lactobacillaceae Yara019 (n = 6), 9) *Bacillus sonorensis* Yara006 (n = 6), and 10) *Rummeliibacillus suwonensis* Yara005 (n = 6). This experiment lasted for 17 d.

### Feed, Water, and Temperature Monitoring

After exposure to PBS or bacteria, the chicks were raised under standard conditions. All chicks were kept in the same room but each group was kept in a separate clean plastic container (total area of each container: 2,368 cm^2^) in the chicken house at the Faculty of Agriculture. Each plastic container was equipped with 2 clean feeders, 2 water bottles and sawdust as bedding for the chicks. Chicks were marked with different colors before the experiment for identification. The room was preheated to 32°C a day before the arrival of chicks to maintain the brooding temperature for chicks (Aviagen, 2018). For the first three days, the chicks were kept under high temperature (32°C) and from the third day onward, the temperature was decreased (30°C) and adjusted according to the behavior and age of chicks. The litter (sawdust) of the chick's containers was changed on a weekly basis. Frequent monitoring and inspection of the chicks was done to check for any abnormalities or pathological conditions. Feed was corn based and contained no antibiotics.

### Sample Collection

For the second experiment fecal samples were collected from each chick individually on d 2, d 7, and d 17 of life in 15 mL tubes containing 5 mL PBS. For fecal sampling individual chicks were placed within a separate container on a clean piece of paper. No fecal samples were collected in the first experiment. On the 7th day of the first experiment and the 17th day of the second experiment, all the chicks were euthanized using CO_2_ and then weighed. Cecal contents were squeezed into tubes with 5 mL PBS and the jejunal contents were collected by using a syringe loaded with PBS to flush the contents out from a piece of the jejunum into tubes with the final PBS volume being 5 mL. All samples fecal, jejunal, and cecal, were immediately frozen in liquid nitrogen and later preserved in −20°C.

### DNA Extraction

DNA was extracted from all samples of both experiments by disruption with 0.1-mm glass beads in the presence of Tris-saturated phenol, following phenol-chloroform extraction, as described by (Stevenson and Weimer, 2007). Briefly, the aqueous fractions were mixed with equal volumes of phenol and separated by centrifugation. This step was repeated twice, following 2 aqueous phase extraction with a 1:1 (vol:vol) mixture of phenol and chloroform, and lastly, 2 aqueous phase extractions with chloroform. The DNA was subsequently precipitated using isopropanol precipitation and suspended in DDW. Afterward the concentration of extracted DNA was measured spectrophotometrically using Nanodrop and each sample was diluted to 10 ng/µL. DNA was kept at 4°C for further analysis.

### 16S rDNA Sequencing

16S rDNA library preparation and sequencing were performed according to the Earth Microbiome Project Protocol (Thompson et al., 2017) using V4 primers 515 Forward (GTGYCAGCMGCCGCGGTAA) and 806 Reverse (GGACTACNVGGGTWTCTAAT). In short, DNA was used as template and the variable region V4 of the 16S rDNA gene was amplified by PCR using universal primers set 515 Forward and 806 Reverse tagged with adapter sequences and unique barcodes. Negative controls for each barcoded forward primer were also included. Thermocycling was performed under the following conditions: 94°C for 3 min, followed by 35 cycles of 94°C for 45 s, 50°C for 60 s, and 72°C for 90 s, and a final step of 72°C for 10 min. Hundred and fifty base pairs paired-end sequencing was performed on an Illumina Miseq platform using a V2 reagent kit by the sequencing unit of the Faculty of Medicine at the Hebrew University of Jerusalem and Hylabs (Rehovot). Sequences were processed and taxonomy was assigned using QIIME2 (Bolyen et al., 2019). Dada2 plugin version 2018.8.0 (Callahan et al., 2016) was used to determine amplicon sequence variants (**ASVs**) using the denoise-paired method. An average of 82.9 ± 8.2% of the raw sequences were retained by Dada2, with an average of 19,381 ± 13,457 reads per sample. ASVs with under 5 reads were discarded. All samples were normalized to 6,000 reads per sample. Taxonomy was assigned using a naive-bayes classifier (Pedregosa et al., 2011) trained on the Greengenes database (McDonald et al., 2012).

### Statistics

Statistical analysis was done by using *t* test in JMP 16 and GraphPad Prism 6. Results are presented as Mean ± SD. Statistical significance was accepted at *P* < 0.05. For Figure 3 multiple comparison was done between the groups using Tukey HSD test in Graph Pad Prism6. Different connecting letters denote the groups that are significantly different.

## Results

### Broiler Colonization

For the first experiment 7 strains of bacteria of diverse phylogeny were chosen. *Phocaeicola plebeius* Yara004 and *Phocaeicola Salanitronis* Yara001 of order Bacteroidales were chosen as Bacteroidales have been shown to successfully colonize layers (Kubasova et al., 2019) and as they are propionate producers (Rios-Covian et al., 2017). *Ligilactobacillus salivarius* Yara018 and *Limosilactobacillus reuteri* Yara020 of order Lactobacillales were chosen as these are species frequently utilized as probiotics (Al-Khalaifah, 2018). *Pediococcus pentosaceus* Yara000 of the same order was also picked as this genus is also utilized in probiotic formulations (Al-Khalaifah, 2018). Finally, two phylogenetically very different bacteria were chosen with the aim of expanding our colonization screen, *Massiliomicrobiota timonensis* Yara022 of order Erysipelotrichales and *Ruminococcus torques* Yara008 of order Clostridiales.

**Figure 1:**
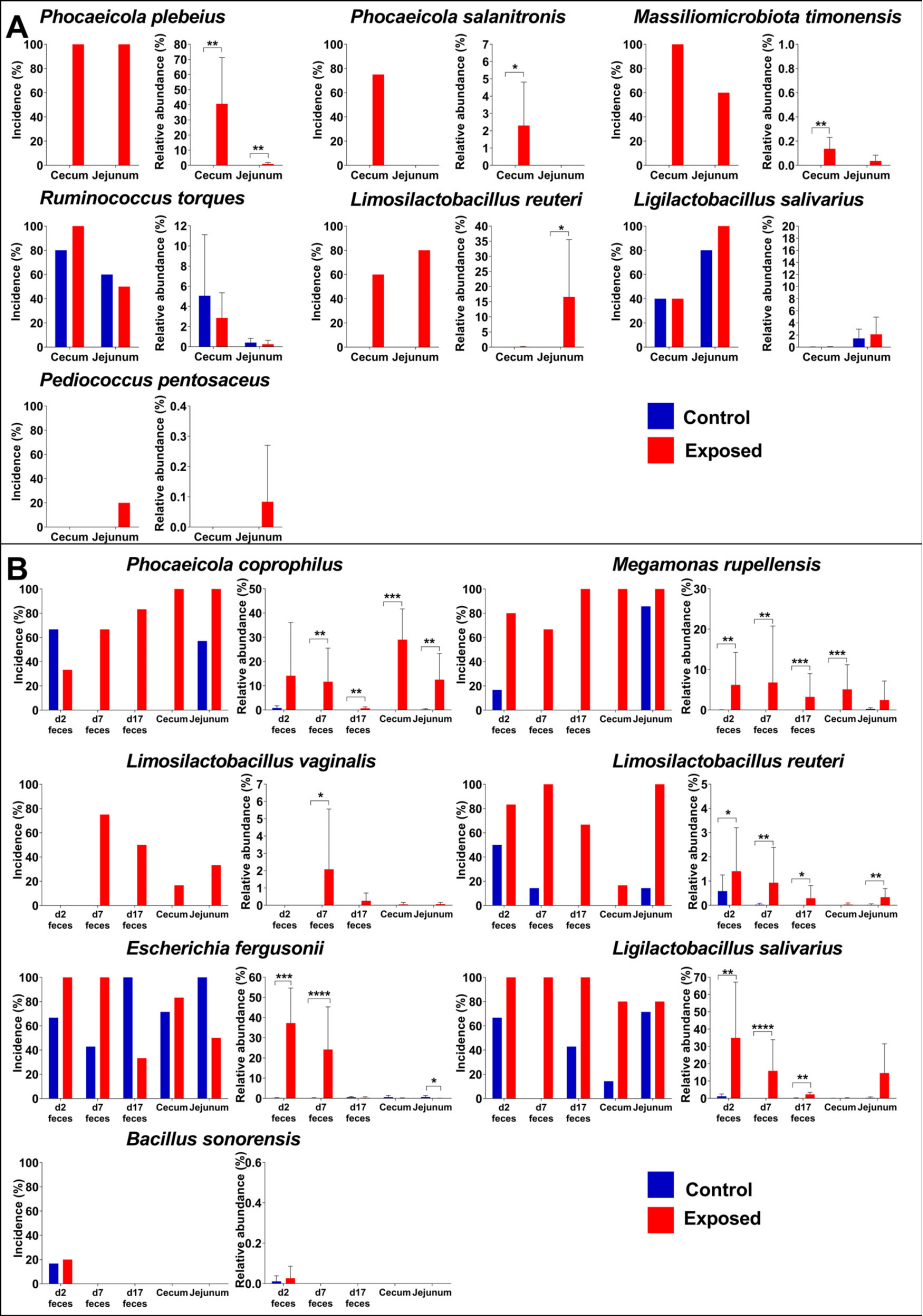
Incidence and relative abundance of bacterial strains given on day of hatch in chicken samples collected on d 2, 7, and 17. Experiment 1 done in broilers (A), experiment 2 in layers (B). For each treatment incidence is shown on the left while relative abundance is shown on the right. Levels in exposed chicks shown in red, in non-exposed control chicks shown in blue. In experiment 1 n = 5 for all groups except for *P. salanitronis* Yara001 in the cecum, *P. plebeius* Yara004 in the jejunum, and *R. torques* Yara008 in the jejunum in which n = 4, and *P. salanitronis* Yara001 in the jejunum in which n = 2. In experiment 2, n = 6 for all except for *L. salivarius* Yara018 for which n = 5 in both cecum and jejunum. Statistical analysis was done by using *t* test in JMP 16 and GraphPad Prism 6. Results are presented as Mean ± SD. * 0.01 ≤ *P* <0.05, ** 0.001 ≤ *P* < 0.01, *** *P* < 0.001, **** *P* < 0.0001.

No differences in final weight of the chicks were found (Supplementary Figure 1A). 16S rDNA sequencing showed variable colonization success (Figure 1A). Both *Phocaeicola* species Yara004 and Yara001 were very good colonizers of newly hatched chicks. *P. plebeius* Yara004 was identified in all treated chicks and accounted for 40.6 ± 30.5% of all cecum bacteria. *P. salanitronis* Yara001 was found in most treated chicks and accounted for 3.1 ± 2.4% of all cecum bacteria. *P. salanitronis* Yara001 was completely absent in jejunum samples. Also, *M. timonensis* Yara022 and *L. reuteri* Yara020 were good colonizers of chicks. *L. reuteri* Yara020 became a prominent member of the jejunum community accounting for 16.6 ± 18.9%. Interestingly, incidence and relative abundance of *L. salivarius* Yara018 and *R. torques* Yara008 treated chicks was high but not different from controls (Figure 1A). While we cannot prove that the background colonizer was the same strain, this is highly possible as the strains utilized were isolated from a hen growing in the same facility. Thus, it can be concluded that for these 2 strains treatment had no effect, likely because the same strain was naturally colonizing the chicks. As expected, *L. salivarius* Yara018 like *L. reuteri* Yara020 colonized preferentially the jejunum. Only one strain, *P. pentosaceus* Yara000, seemed to be a very poor colonizer of chicks in this experiment (Figure 1A), as its incidence in treated chicks was low. Thus, these results show that artificially introduced bacteria were variably capable of colonizing newly hatched broilers.

### Layer Colonization

For the second experiment 9 different bacterial strains were picked. The *L. salivarius* Yara018 and *L. reuteri* Yara020 strains used in the boiler experiment were used in this experiment as well. Two other Lactobacillales strains, a *Limosilactobacillus vaginalis* Yara017 and an unclassified Lactobacillaceae Yara019 were also included. A different Bacteroidales, *P. coprophilus* Yara003, a different Clostridiales, *Megamonas rupellensis* Yara012, 2 strains of order Bacillales, *Bacillus sonorensis* Yara006 and *Rummeliibacillus suwonensis* Yara005, and an Enterobacterales, *Escherichia fergusonii* Yara015 were chosen to increase the phylogenetic diversity tested.

No differences in final weight of the chicks were found (Supplementary Figure 1B). 16S rDNA sequencing again showed variable colonization success (Figure 1B). *P. coprophilus* Yara003 colonized all treated chicks and became a major component of both the cecum and jejunum microbiota accounting for 29.04 ± 12.7% and 12.5 ± 10.7%, respectively. *M. rupellensis* Yara012*, L. reuteri* Yara020, and *L. salivarius* Yara018 were also good colonizers of newly hatched chicks. *L. vaginalis* Yara017, was able to colonize newly hatched chicks but considering the low incidence and relative abundance, was not a good colonizer compared to the previous four. *E. fergusonii* Yara015 incidence and relative abundance were high compared to controls in d 2 and d 7 fecal samples but later on were not different from controls. Both *L. salivarius* Yara018 and *L. reuteri* Yara020 showed a preference for jejunal colonization. *B. sonorensis* Yara006 had a low incidence in treated chicks. Furthermore, this bacterium was not identified in any of the samples on d 17. Thus, colonization of *B. sonorensis* Yara006 failed in our experiment. Last, sequences of the unclassified Lactobacillaceae Yara019 were not identified at all, and most chicks treated with *R. suwonensis* Yara005 had become sick and had to be euthanized. Dissection of these chicks showed bloated intestinal tracts filled with gas. While it is possible that *R. suwonensis* Yara005 is directly responsible for this result, it is also possible that by chance a pathogen infected this group of chicks. Furthermore, we did obtain a few gut samples from this group of chicks and were unable to identify *R. suwonensis* Yara005 or any clearly identifiable bacterial pathogen. Thus, it seemed that *R. suwonensis* Yara005 did not colonize chicks in this experiment. It should be noted that we did not spurolate *R. suwonensis* Yara005 or *B. sonorensis* Yara006 and it is possible that this is the reason for their failed colonization. To conclude, also in layers and in a longer 17-d experiment we found variable colonization success.

### Analysis of Colonization Through Fecal Samples

In the second experiment fecal samples were collected on d 2, 7, and 17. This was to examine if fecal samples were a viable alternative to internal, jejunal, and cecal content samples, with the aim of reducing the number of animals in future experiments. In regards to incidence on d 17, fecal samples were generally in agreement with the incidence identified from internal samples (Figure 1B). Unsurprisingly, fecal samples poorly represented the relative abundance in internal organs. This was especially clear for *P. coprophilus* Yara003 in which the relative abundance in d 17 fecal samples was much lower than internal samples. Fecal samples did allow observations into colonization dynamics. For example, *P. coprophilus* Yara003 incidence in treated chicks increased over time whereas *L. salivarius* Yara018 colonized all treated chicks already on d 2. Furthermore, differences in incidence and relative abundance on d 2 and 7 of *E. fergusonii* Yara015 in treated chicks compared to controls showed that artificial exposure had an effect. This effect is lost for *E. fergusonii* Yara015 when examining d 17 fecal samples and internal samples. Thus, fecal samples are a viable alternative to internal samples for the aim of identifying colonization of newly hatched chicks.

### Effects on the Microbiota

**Figure 2:**
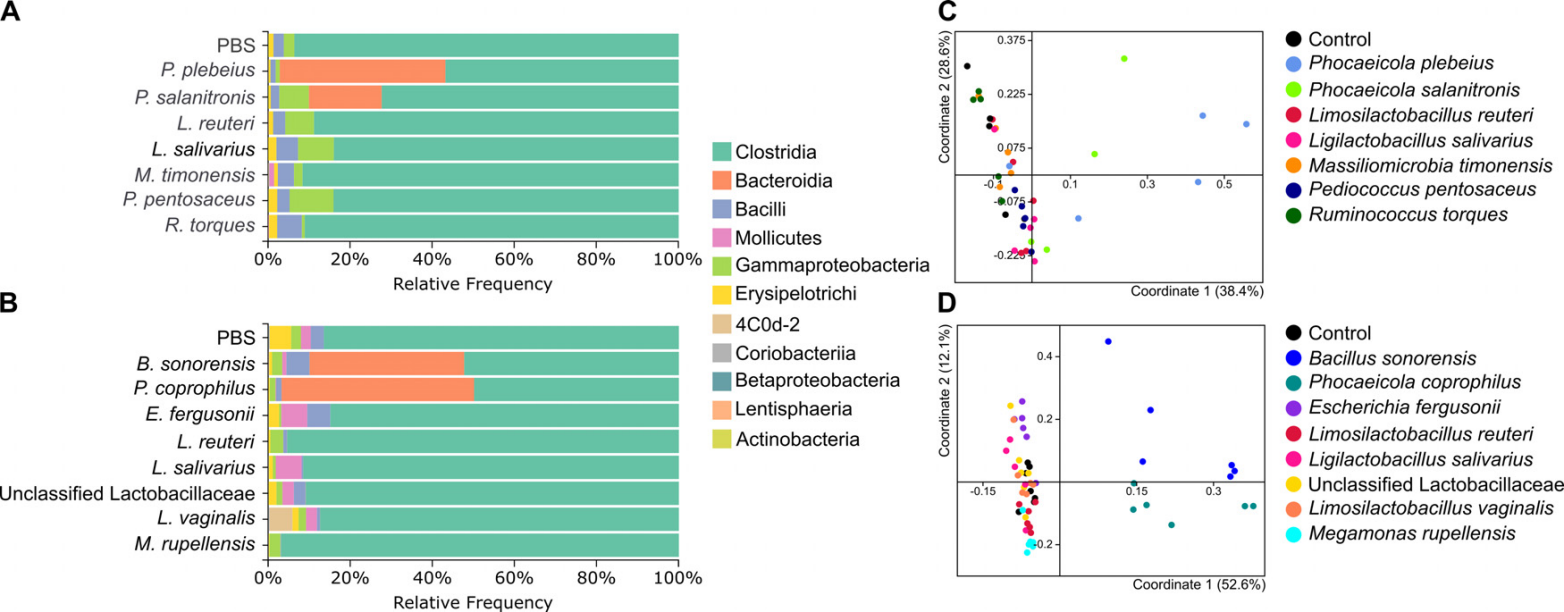
Community structure of cecum microbiota of chicks exposed to different bacteria. Experiment 1 done in broilers (A, C), experiment 2 in layers (B, D). (A, B) Taxonomic composition of the ceca of different groups at the class level. (C, D) PCoA based on weighted UniFrac distances at the ASV level.

To understand if artificial colonization by single strains of bacteria modulated the microbiota, all 16S rDNA sequencing data was analyzed by weighted UniFrac. A PcoA based on weighted UniFrac distances showed that in both experiments chicks colonized with Bacteroidales strains had a different cecum community (Figures 2A–D). In experiment 2, the microbiota of chicks that were exposed to *Bacteroidales* strains was significantly different from that of control chicks (PERMANOVA, test with 999 permutations and Benjamini-Hochberg FDR correction, *P* = 0.009); while significance is not reached in experiment 1, probably due to high variability within the treated groups. Interestingly, this was true whether chicks were deliberately exposed to a Bacteroidales strain which colonized them, such as *P. coprophilus* Yara003*, P. plebeius* Yara004, or *P. salanitronis* Yara001, or when a Bacteroidales strain colonized a group of chicks unintentionally, as has happened for the chicks exposed to *B. sonorensis* Yara006. It should be noted that because of lack of repeats it is unknown if there is a connection between exposure to *B. sonorensis* Yara006 and Bacteroidales colonization. It should also be noted that the Bacteroidales strain that grew in the *B. sonorensis* Yara006 treated chicks was not one of the strains used to treat other groups of chicks. In any case, colonization by Bacteroidales resulted in a large change in community structure, mainly because the Bacteroidales strains themselves became major components of the community.

An analysis of weighted and unweighted UniFrac distances between treatment groups and the control group found in general that cecum communities were different on d 17 by both weighted and unweighted UniFrac, as well as d 2 fecal samples by weighted UniFrac, and d 7 fecal samples by unweighted UniFrac (Supplementary Figure 2). Because these differences were found across the board and also in treatment groups in which it seemed that the bacterial strain used for exposure failed to colonize, it is likely that these differences possibly represent general diversification of these bacterial communities, coupled with a cage effect, rather than a specific effect of exposure to our bacterial strains. However, it should be noted that *Bacteroidales*-treated groups had a higher pseudo-F value (21.3–22.3) than that of other groups (2.4–6.6), indicating a more pronounced difference in these groups (PERMANOVA test between each treatment and the control with 999 permutations and Benjamini-Hochberg FDR correction).

**Figure 3:**
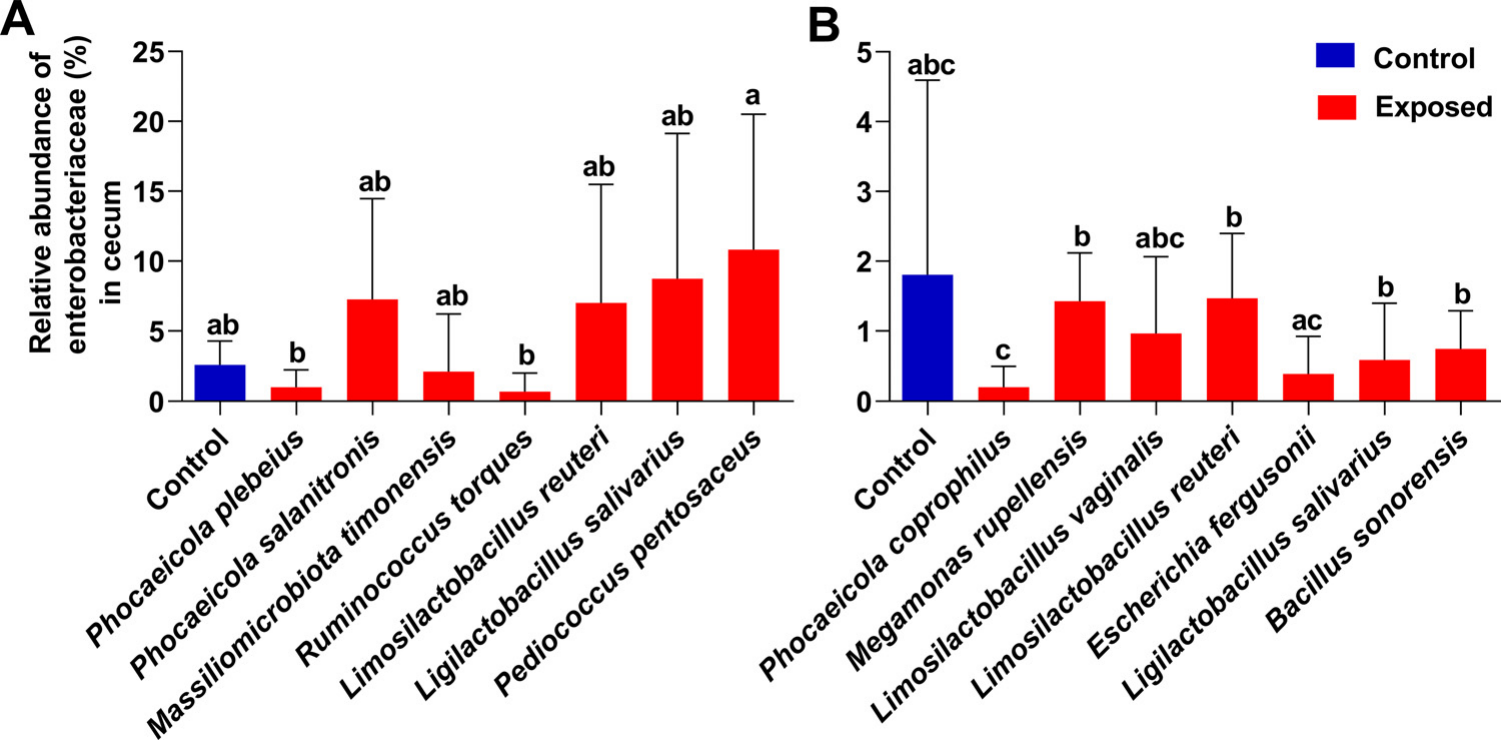
Relative abundance of family Enterobacteriaceae in cecum samples. Experiment 1 done in broilers (A), experiment 2 in layers (B). Levels in exposed chicks shown in red, in non-exposed chicks shown in blue. In experiment 1 n = 5 for all groups except for *P. salanitronis* Yara001. In experiment 2, n = 6 for all except for *L. salivarius* Yara018 for which n = 5. For statistical analysis multiple comparison was done between the groups using Tukey HSD test in Graph Pad Prism6. Results are presented as Mean ± SD. Different connecting letters denote the groups that are significantly different.

Last, the levels of the family Enterobacteriaceae in the different treatment groups were examined. This is because a number of potential pathogens, including *Salmonella* and pathogenic *E. coli* are members of this family. We hypothesized that members of Enterobacteriaceae have common environmental preferences, for example, preferred nutrients or gut binding sites, and that reduction in levels of Enterobacteriaceae in a treatment group might imply a reduced ability of pathogens from this family to successfully colonize treated chicks. A large variation in Enterobacteriaceae levels in controls resulted in non-statistically significant differences compared to the control group (Figure 3). However, when examining the cecal levels of Enterobacteriaceae in all treatment groups, in the first experiment chicks treated with either *P. plebeius* Yara004 or *R. torques* Yara008 had less Enterobacteriaceae (Figure 3A). In the second experiment lower levels of Enterobacteriaceae were found in the ceca of *P. coprophilus* Yara003 chicks (Figure 3B). Thus, some chick colonizers were possibly affecting Enterobacteriaceae levels.

## DISCUSSION

The colonization ability, or lack thereof, is part of the mechanism of action of probiotic strains, and in the absence of this data it is impossible to understand how probiotics work and how to improve them. For example, it is not known whether continued treatment is required, as initial inoculation may be sufficient to achieve the desired effect. Likewise, it is possible that the transient presence of a bacterial strain during key early developmental steps may be sufficient to modulate subsequent development without further bacterial involvement. Very little information on the colonization ability of probiotics is found in the literature. Here, we screened 14 bacterial strains for their colonization ability in both broilers and layers, characterized their colonization of the jejunum, cecum, and feces, measured their colonization up to d 17 postinoculation, and characterized their effects on the gut microbial community profile.

In this study, the colonization ability of 14 bacterial strains was examined, including strains of species already used in commercial probiotic products and other bacteria not yet tested as probiotics. It was found that 10 bacterial strains were successful colonizers of chickens. Thus, this study confirms the results of a previous studies showing that Bacteroidales and Negativicutes strains colonize newly hatched chicks well (Kubasova et al., 2019; Zenner et al., 2021). However, Lactobacillales strains used in both studies either failed to colonize or were not different in terms of incidence and relative abundance from untreated controls. Here, a clear colonization signal was found also for *L. vaginalis* Yara017*, L. reuteri* Yara020, and *L. salivarius* Yara018 colonization. Thus, at least some Lactobacillales strains are not efficient natural colonizers of newly hatched chicks and can benefit from artificial exposure. Interestingly, in the broiler experiment described here *L. salivarius* Yara018 was also found in untreated controls, whereas in the layer experiment it was absent from controls. It is not known if this happened by chance, or because of a difference between broilers and layers. However, this result shows that artificial exposure can compensate natural colonization variability, as well as explain the difference in the results of this work compared to the 2 published works. Last, 4 strains used in this study failed to colonize newly hatched chicks. This failure could be a result of inoculum preparation, for example, unsporulated inoculum in preparation for inoculation, or experimental conditions, and does not have to be the result of the inability of these strains to colonize newly hatched chicks. Indeed, as these strains were isolated from adult hens, it is likely that they can at the very least colonize adults. It should also be noted that the ability of any species to colonize chicks is strain specific and thus these results mainly show the potential of strains of these species to colonize newly hatched chicks.

The strains used here could be grouped into 4 groups. Strains which were good natural colonizers, in that nontreated chicks were also efficiently colonized, such as *R. torques* Yara008 which had an incidence of 80% in control chicks. Similarly, *L. salivarius* Yara018 in the first experiment had similar incidence and relative abundance levels in treated and control chicks. Artificial exposure is likely to have a minimal effect on bacteria of this group which naturally colonize chicks, with the caveat that natural colonization might be inconsistent. The second group includes strains in which a single artificial exposure treatment resulted in very high colonization. This group includes the 3 Bacteroidales strains, *M. rupellensis* Yara012*, M. timonensis* Yara022*,* and *L. reuteri* Yara020. A single treatment of bacterial strains of this group is likely enough to ensure colonization and repeated exposure is likely redundant. The third group includes all the strains in which it was not clear that exposure resulted in any effect on gut bacterial levels. This group includes the strains in which colonization was very low overall, such as *P. pentosaceus* Yara000 and *B. sonorensis* Yara006*.* If these strains have any positive effect on the host, then continuous administration will likely be required, unless just a single passage through the intestinal system is enough to create signals which modify developmental outcomes. The last group includes only *L. vaginalis* Yara017. A single exposure event clearly resulted in colonization. However, overtime incidence dropped. Colonization by this strain would likely benefit from continuous exposure. Thus, characterization of the levels of used strains after a single exposure is a good tool to understand if a single exposure is sufficient or if continuous application is required for a specific probiotic strain.

The first group described above, in which artificial exposure had no effect because of natural high background colonization, is of particular interest. This is an important observation since many probiotic strains originate from poultry. If these strains are already present in chicks, it is unclear whether the administration of more bacteria can increase their levels in the gut, or whether this will have any effect on the function of the strain or microbiota. Furthermore, if natural colonization is variable this might explain why some experiments work and others do not and why large scale experiments would show a small effect. Interestingly, one such strain in our study is *L. salivarius* Yara018, which as a species is commonly used as a probiotic (Chaves et al., 2017). It is clear that this is entirely dependent on the natural prevalence of *L. salivarius* Yara018 and chance, as is exemplified by the different outcomes in control chicks in the 2 experiments, but other studies characterizing early colonizers have also identified this species (Johnson et al., 2018; Ngunjiri et al., 2019). It is also possible that the presence of these bacteria in the controls is due to cross-contamination. However, since other strains used in this study were not found in untreated birds, we are confident that this is due to natural background colonization.

This study shows that different bacteria had different colonization preferences. *P. plebeius* Yara004 and *P. salanitronis* Yara001 were clearly cecum colonizers, with *P. salanitronis* Yara001 levels in the jejunum found to be below the detection limit. Meanwhile, *L. reuteri* Yara020 and *L. salivarius* Yara018 had a very strong preference for the jejunum. Thus, it is clear that at the very least these 2 different intestinal sites should be sampled to determine colonization success. This also implies that different bacterial strains might have very different effects on the host depending on the site of colonization. Thus, characterizing colonization at different gut sites might help to elucidate the mechanism of action of different probiotic strains. For example, a strain that colonizes or affects the cecum microbiota is more likely to affect short-chain fatty acid levels than a strain that is strictly a jejunum colonizer.

In this study, the colonization in newly hatched chicks of both layer and broiler breeds was characterized. In both cases, it was found that Bacteroidales strains were able to efficiently colonize chicks and in both they became a major component of the cecum community. Furthermore, for both breeds, colonization by *L. reuteri* Yara020 and *L. salivarius* Yara018 was identified. Therefore, at least in the broader view, the interaction of gut bacteria with newly hatched chicks of layer and broiler breeds appears to be similar, implying that probiotics will be interchangeable for both.

In the second experiment, fecal samples were also collected. The fecal samples were collected on d 2, 7, and 17 of life. This last fecal sample was collected on the same day that the birds were euthanized and the internal organs were sampled. As expected, the fecal samples did not represent the relative abundance of the treated strain in the internal organs well. Nevertheless, fecal samples were a good proxy when analyzing the ability of a particular bacterial strain to colonize newly hatched chicks. Fecal samples also allowed identification of background colonization of untreated chicks. Last, it seems that fecal samples allowed the characterization of the dynamics of colonization, though it could not be verified in this study because we lack internal samples at d 2 and 7. For example, in *P. coprophilus* Yara003 treated chicks, fecal samples reveal a gradual rise in incidence overtime, which cannot be inferred by examining incidence in internal organs on d 17. Thus, fecal samples allow continued sampling of the same specific individuals, possibly allow following colonization dynamics, and allow a reduction in the number of birds required for each experiment.

The effects of the different treatments on the development of the microbiota were also investigated. Differences in microbiota composition were identified in all treated groups. However, because each treated group was housed in a separate container and even the groups in which there was no apparent colonization by the treated bacterial strains were still different, it is likely that these differences were a result of cage effect. However, analysis utilizing weighted UniFrac showed that chicks colonized by Bacteroidales strains, either by artificial treatment or incidentally by background colonization, had a unique cecum microbiota that were markedly different from other chick groups. Thus, as expected, colonization by bacteria which became very large components of the microbiota modulated microbiota composition.

Last, the levels of family Enterobacteriaceae in the different treatment groups were measured. This family includes a number of important pathogens, including *Salmonella* and pathogenic *E. coli*. It was hypothesized that a specific treatment that reduces Enterobacteriaceae levels might similarly affect the relevant pathogens because some environmental requirements are conserved among closely related bacteria. For example, a reduction in specific available nutrients or gut binding sites might hypothetically affect all Enterobacteriaceae. While a few treatments were found to reduce Enterobacteriaceae levels, especially promising was the fact that both *P. coprophilus* Yara003 and *P. plebeius* Yara004, which became major components of the cecum, reduced Enterobacteriaceae levels. Further research is required to determine whether these or other strains used here could inhibit pathogen colonization.

## CONCLUSIONS

Here the colonization ability of 14 diverse strains of bacteria was studied. We found most of them, including Lactobacillales strains which are commonly used as probiotics, were able to colonize after a single treatment on the day of hatch. Treatment with some strains had a lesser effect, because of high background colonization. Only 4 of the 14 strains used did not colonize newly hatched chicks in this study. These results demonstrate the importance of characterizing the levels of probiotic strains in treated chickens. Evaluation of the levels of probiotic strains could show that a single treatment is sufficient as it results in colonization or that the strain is acquired naturally, and thus artificial exposure might be redundant. Even if the probiotic strain is unable to colonize chickens, noting this fact could lead to the identification of similar strains conferring the same function but able to colonize the chicken, potentially leading to more significant beneficial effects.

It was also found that a single exposure on the day of hatch caused alterations in the gut microbiota that lasted for at least 17 d. This included very large changes in the composition of the microbiota in the *P. coprophilus* Yara003 treated chicks. While that specific experiment was performed on layers, these findings are even more relevant to broilers, as 17 d represent a third of their commercial lifespan. Thus, it is possible to modulate the microbiota effectively by a single exposure at the day of hatch. Further research is required to understand the effect of such a modification on the functions of the microbiota and the development of the chick.

## Appendix Supplementary materials

Image, application 1

Supplementary Figure 1: Body weight of control and bacteria exposed chicks at the end of the first experiment utilizing broilers, n=9 (A), and the end of the second experiment utilizing layers n=7 for control and n=6 for all other groups except *L. salivarius* that had n=5 (B) recorded on day 7 and day 17 respectively. No statistical significance (P < 0.05) was found utilizing t-test in Graph Pad Prism 6. Results are presented as Mean ± SD. Image, image 2

Supplementary Figure 2: Weighted and unweighted UniFrac distances of different groups from the control group (PBS). Experiment 1 done in broilers (A), experiment 2 in layers (B). PERMANOVA test between each treatment and the control with 999 permutations and Benjamini-Hochberg FDR correction; * *p* < 0.05, ** *p* ≤ 0.01. Image, image 3
