# Supplementary material for: Characterizing the chicken gut colonization ability of a diverse group of bacteria
Source: Poult Sci. 2022 Aug 15;101(11):102136. doi: 10.1016/j.psj.2022.102136 (PMC9508342; doi:10.1016/j.psj.2022.102136)
Supplement: Supplementary file 1 [file mmc1.docx]

| Strain name | Blast hit | | Isolation conditions | Isolation and growth media |
| --- | --- | --- | --- | --- |
|  | Scientific name | % Identity |  |  |
| Yara000 | *Pediococcus pentosaceus* | 99.68 | Anaerobic | YCFA |
| Yara001 | *Phocaeicola salanitronis* | 97.17 | Anaerobic | YCFA |
| Yara003 | *Phocaeicola coprophilus* | 99.27 | Anaerobic | YCFA |
| Yara004 | *Phocaeicola plebeius* | 98.29 | Anaerobic | YCFA |
| Yara005 | *Rummeliibacillus suwonensis* | 99.54 | Aerobic | YCFA |
| Yara006 | *Bacillus sonorensis* | 99 | Aerobic | YCFA |
| Yara008 | *Ruminococcus torques* | 94.69 | Anaerobic | YCFA |
| Yara012 | *Megamonas rupellensis* | 96.49 | Anaerobic | YCFA |
| Yara015 | *Escherichia fergusonii* | 98.89 | Aerobic | YCFA |
| Yara017 | *Limosilactobacillus vaginalis* | 98.84 | Aerobic | MRS |
| Yara018 | *Ligilactobacillus salivarius* | 99.09 | Aerobic | MRS |
| Yara019 * | *Ligilactobacillus salivarius* | 93.01 | Aerobic | MRS |
| Yara020 | *Limosilactobacillus reuteri* | 98.39 | Aerobic | MRS |
| Yara022 | *Massilimicrobiota timonensis* | 97.59 | Anaerobic | YCFA |

**Table S1:** information on the isolated strains used in this study to inoculate newly hatched chicks. Strains were isolated from a cecum content sample of a healthy 55-week old Ross broiler breeder. Isolation conditions and media are specified in the table. The 16S rRNA gene of each isolate was sequenced using the universal primers 27F and 1492R (Heuer et al., 1997). Taxonomic annotation for each isolate was assigned by comparing their 16S sequence to the NCBI 16S rRNA database (Sayers et al., 2022) using BLAST (Altschul et al., 1990). Best BLAST hit is presented in the table. All E-values were 0.0.

* The best BLAST hit for isolate Yara019 was *Ligilactobacillus salivarius*, with 93.01% identity, therefore it is referred to in this study as Unclassified *Lactobacillaceae*.

References:

Altschul, S.F., Gish, W., Miller, W., Myers, E.W., Lipman, D.J., 1990. Basic local alignment search tool. Journal of Molecular Biology 215, 403–410. https://doi.org/10.1016/S0022-2836(05)80360-2

Heuer, H., Krsek, M., Baker, P., Smalla, K., Wellington, E.M., 1997. Analysis of actinomycete communities by specific amplification of genes encoding 16S rRNA and gel-electrophoretic separation in denaturing gradients. Applied Environmental Microbiology 63, 3233–3241. https://doi.org/10.1128/aem.63.8.3233-3241.1997

Sayers, E.W., Bolton, E.E., Brister, J.R., Canese, K., Chan, J., Comeau, D.C., Connor, R., Funk, K., Kelly, C., Kim, S., Madej, T., Marchler-Bauer, A., Lanczycki, C., Lathrop, S., Lu, Z., Thibaud-Nissen, F., Murphy, T., Phan, L., Skripchenko, Y., Tse, T., Wang, J., Williams, R., Trawick, B.W., Pruitt, K.D., Sherry, S.T., 2022. Database resources of the national center for biotechnology information. Nucleic Acids Research 50, D20–D26. https://doi.org/10.1093/nar/gkab1112
