# Supplementary figures and images for: Characterizing the chicken gut colonization ability of a diverse group of bacteria
Source: Poult Sci. 2022 Aug 15;101(11):102136. doi: 10.1016/j.psj.2022.102136 (PMC9508342; doi:10.1016/j.psj.2022.102136)

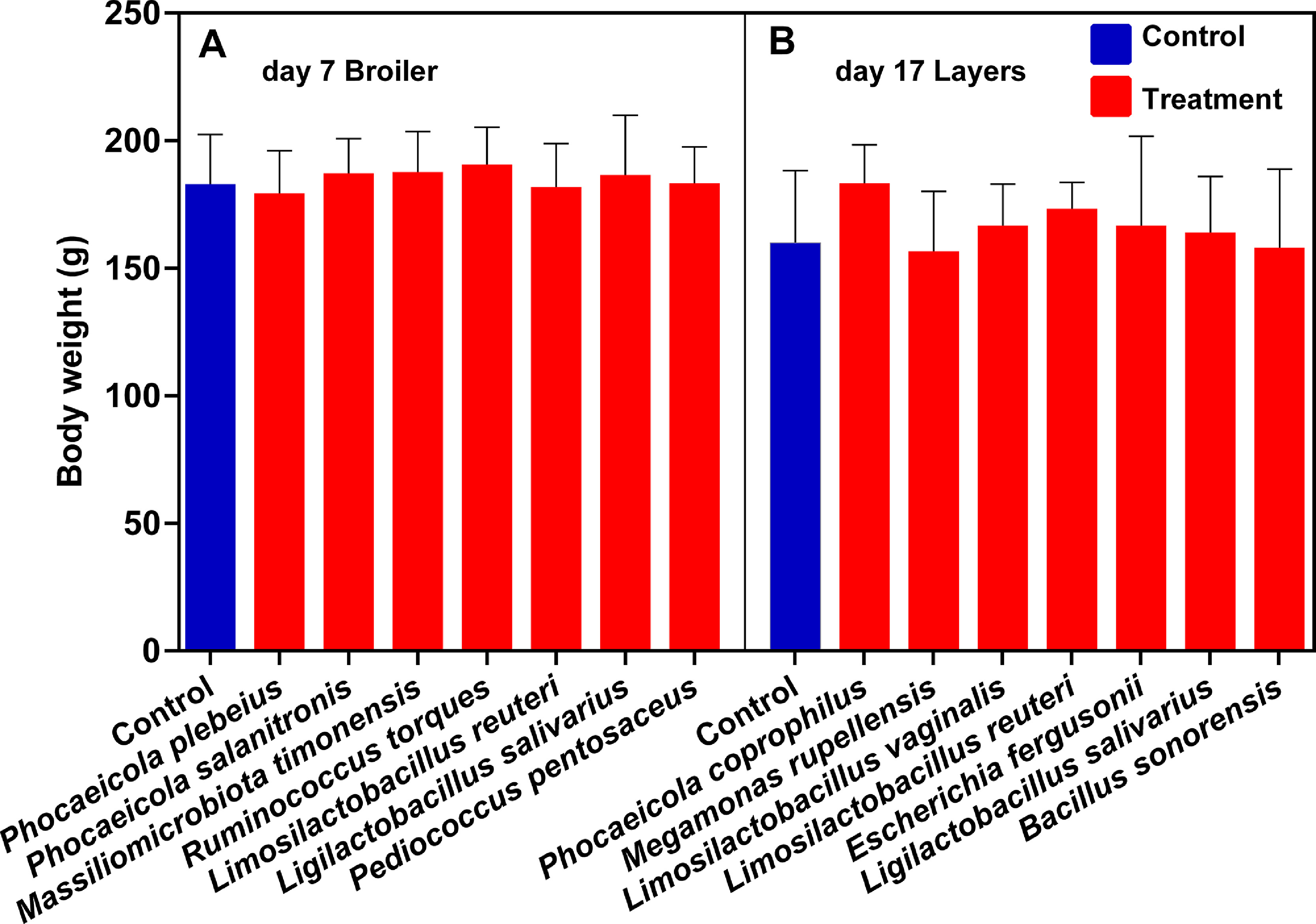

Supplement: Supplementary file 2 — Supplementary Figure 1: Body weight of control and bacteria exposed chicks at the end of the first experiment utilizing broilers, n=9 (A), and the end of the second experiment utilizing layers n=7 for control and n=6 for all other groups except L. salivarius that had n=5 (B) recorded on day 7 and day 17 respectively. No statistical significance (P < 0.05) was found utilizing t-test in Graph Pad Prism 6. Results are presented as Mean ± SD. [file mmc2.jpg]

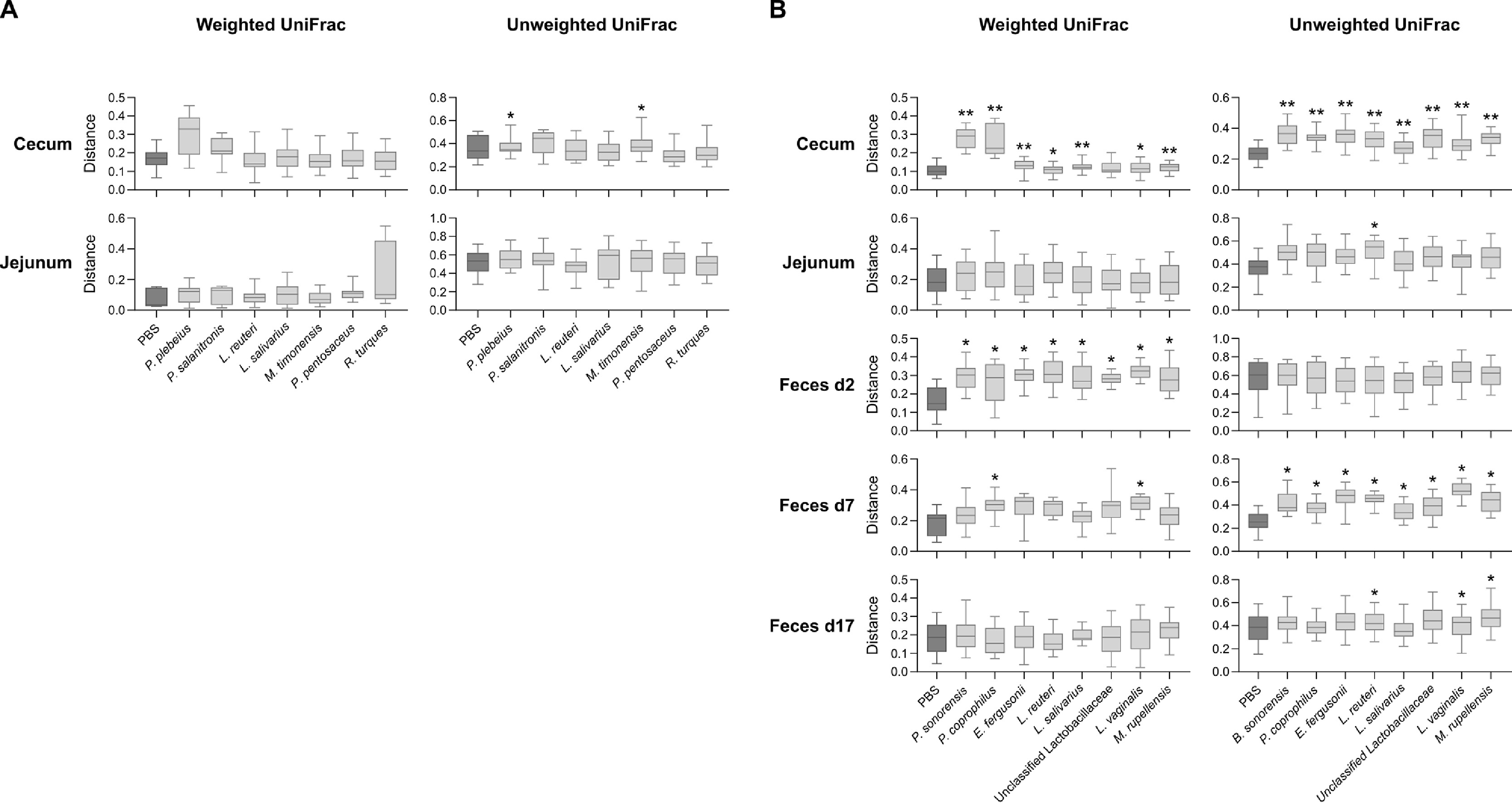

Supplement: Supplementary file 3 — Supplementary Figure 2: Weighted and unweighted UniFrac distances of different groups from the control group (PBS). Experiment 1 done in broilers (A), experiment 2 in layers (B). PERMANOVA test between each treatment and the control with 999 permutations and Benjamini-Hochberg FDR correction; * p < 0.05, ** p ≤ 0.01. [file mmc3.jpg]
